# Barriers of Doctors and Patients in Starting Insulin for Type 2 Diabetes Mellitus

**DOI:** 10.7759/cureus.18263

**Published:** 2021-09-25

**Authors:** Haider A Alidrisi, Ali Bohan, Abbas A Mansour

**Affiliations:** 1 Diabetes and Endocrinology, College of Medicine, University of Basrah, Basrah, IRQ

**Keywords:** type 2 diabetes mellitus (t2dm), doctors' barriers, patients' barriers, insulin injection, insulin

## Abstract

Background

Management of patients with type 2 diabetes mellitus (T2DM) may involve insulin therapy. However, this treatment may be avoided or delayed by physicians or patients due to the presence of certain barriers. This study aimed to evaluate the barriers to initiating insulin therapy for both physicians and patients with T2DM.

Method

This was a cross-sectional, questionnaire-based study. Data related to the physicians’ personal and professional experience were collected, and 15 barriers to initiating insulin therapy were scored by each physician on a four-point Likert scale. Also, the patients’ general data were collected, including previous insulin experience, discontinuation reason, and willingness to start insulin therapy if indicated. Twenty-one other barriers were examined with yes/no questions as well.

Results

For physicians, the patient's treatment compliance, motive, dependence on others for insulin therapy, hypoglycemia, socioeconomic status, occupation, and lack of follow-up were the most highly ranked barriers to initiating insulin therapy. A history of insulin use was reported in 42 (20.7%) patients, 31 of whom had decided to discontinue insulin therapy themselves (73.8%). The three most common reasons for discontinuing insulin therapy among patients were deterioration of T2DM and causing complications, hypoglycemia, and needle injections. Based on the findings, 99 (48.8%) patients were willing to start insulin therapy, if indicated. The family history of insulin therapy was positively correlated with the patient’s willingness to start insulin. On the other hand, it was negatively correlated with a low educational level and some barriers to insulin therapy, such as fear of death, dependence on others, the difficulty of carrying insulin while traveling, follow-up challenges, the difficulty of dosing accuracy, the difficulty of keeping insulin, inconveniences in daily life, considering insulin as the last resort, the deterioration of T2DM with insulin, and social stigma.

Conclusion

The physicians believed that the barriers to initiating insulin therapy were mainly related to the patient's attitudes and thoughts about this therapy. While hypoglycemia and weight gain are well-known side effects of insulin therapy, the most important patient-related barriers to insulin therapy were related to its impact on the patient’s social life and misperceptions about the side effects of insulin.

## Introduction

The prevalence of type 2 diabetes mellitus (T2DM) in 2019 in the Middle East was 12.2% and is estimated to increase to 15.7% by 2045 [1]. In Iraq, the prevalence of T2DM in 2018 ranged from 8.5% to 13.9% [2]. Generally, T2DM can be managed by one or a combination of strategies, including lifestyle modification, insulin administration, or use of anti-diabetic medications. These therapies, along with glucose monitoring, lead to a good glycemic control [3].

Intensive glycemic control from the time of diagnosis reduces the risk of microvascular complications, myocardial infarction, and all-cause mortality in T2DM patients [4]. In these patients, six years after diagnosis, the insulin secretion drops from 50% to less than 25%; therefore, many patients will need insulin therapy to achieve their target glycemic control [5]. In this regard, in a local retrospective study on 12,869 patients with T2DM, more than half of these patients were on insulin therapy [6].

The initiation of insulin therapy may be delayed in clinical practice for many reasons. The most important factor is a delay in treatment intensification despite poor glycemic control, known as clinical inertia. Clinical inertia is a result of the interaction of different barriers for patients, physicians, and healthcare systems [7]. Physician-related barriers to initiating insulin therapy for T2DM patients vary by country, mainly depending on both the healthcare system and culture [8-9]. Physicians may delay insulin therapy until it is absolutely indicated, i.e., after other therapies have failed to maintain the target glycemic control [8].

The physician-related barriers may include their knowledge of updated guidelines, the experience of insulin therapy, beliefs and attitudes toward insulin and diabetes management, side effects of insulin therapy (e.g., hypoglycemia and weight gain), and finally, perceptions about the patients’ attitudes toward insulin therapy [8-11]. On the other hand, patient-related barriers include concerns about hypoglycemia and weight gain, negative effects of therapy on occupation and social life, injection difficulties, perceptions of personal failure in self-management, and effectiveness of therapy [9, 11-12]. Therefore, the present study aimed to investigate the barriers to insulin therapy among physicians and patients with T2DM.

## Materials and methods

This cross-sectional, questionnaire-based study was carried out from February 2019 to December 2020 in Basrah, Southern Iraq. Physicians and non-insulin-treated patients with T2DM were included. Verbal consents were taken from the participants after explaining the aim of the study in accordance with the ethical standards of the Faiha Specialized Diabetes Endocrine and Metabolism Center (FDEMC) Research Committee, from which the ethical approval was obtained (ref #56/35/23), and with the 1964 Declaration of Helsinki and its later amendments or comparable ethical standards. 

Physician-related barriers

A random sample of 45 physicians who were involved in the care of patients with T2DM participated in this study. Those physicians were working in FDEMC, which is a tertiary care center, three general hospitals, and three primary care centers. Their personal information, including age and gender, was collected. Besides, information about the physicians’ specialty and professional experience was obtained, including their specialty (endocrinologist, internist, or family physician), certification duration, and number of T2DM patients managed per week. 

Next, each physician was asked to rate 15 barriers to insulin therapy, based on a four-point Likert scale (1: Strongly disagree, 2: Disagree, 3: Agree, and 4: Strongly agree). These barriers included the patient’s age, treatment compliance, dependence on others for insulin therapy, the difficulty of keeping insulin, hypoglycemia, insulin costs, insulin unavailability, inadequate consultation time, lack of follow-up, the patient’s escape, occupation, the patient’s motive, socioeconomic status, comorbidities, and weight gain.

Patient-related barriers

A random sample of 203 patients with T2DM who were currently non-insulin treated and under the care of the physicians who participated in the study. The general information of each patient, including age, gender, marital status, type of residence (urban or rural), occupation (employed, self-employed, or unemployed), and educational level (illiterate, primary school, secondary school, or college), was collected in this study. Also, their clinical information included the T2DM duration, family history of T2DM, family history of insulin-treated T2DM, diabetic retinopathy, diabetic neuropathy, chronic kidney disease (CKD), hypertension, dyslipidemia, cardiovascular disease (CVD), ischemic heart disease, cerebrovascular accident, and peripheral vascular disease. Next, the patients were verbally asked to answer the following questions related to insulin therapy:

Were you previously treated with insulin?

If yes, was it the physician’s decision or your decision to discontinue insulin therapy?

Why did you decide to discontinue insulin therapy?

If your physician suggests insulin therapy as the only possible treatment to control diabetes at this time, will you start it?

Next, each patient was asked to answer yes/no questions for each of the barriers to determine if they affected the patient’s decision to initiate insulin therapy. These barriers included being alone, fear of death, dependence on others, insulin costs, dependence on others for insulin injection, injection difficulties, difficulty carrying insulin while traveling, difficulty of dosing accuracy, follow-up challenges, difficulty in keeping insulin, hypoglycemia, inconveniences in daily life, considering insulin therapy as the last resort, deterioration of diabetes with insulin, job circumstances, lack of trust in physicians, needle injections, negative attitudes of others, short consultation time, social stigma, and weight gain.

Statistical analysis

Data were analyzed using Statistical Product and Service Solutions (SPSS), version 26.0 (IBM SPSS Statistics for Windows, Armonk, NY). Categorical variables were summarized as number (N) and percentage (%), while continuous variables were presented as mean ± standard deviation (SD). To analyze the effects of patients’ general and clinical characteristics on their willingness to start insulin therapy, a Chi-square test was used, and the odds ratio (OR) and 95% confidence intervals (95% CI) were measured. The Friedman test and Kendall’s W test were also used for comparing the physicians’ scores of barriers, summarized as mean ± standard error (SE) in an error bar chart. A P-value less than 0.05 was considered statistically significant.

## Results

Physician-related barriers

**Figure 1:**
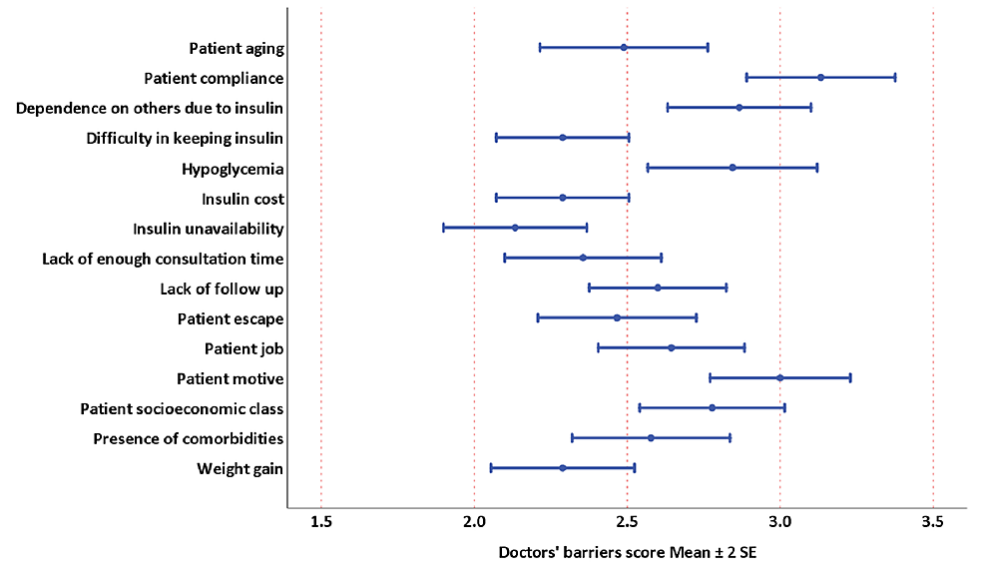
Distribution of the doctors’ responses to different barriers to initiate insulin in their patients Friedman test P-value < 0.0001, Kendall’s W test 0.14, P-value < 0.0001 SE: standard error

The physicians’ general characteristics are summarized in Table 1. There was a significant difference in the physicians’ scoring of barriers to insulin therapy for patients (Figure 1). The most highly ranked barriers to insulin therapy by physicians included the patient’s compliance, patient’s motive, dependence on others for insulin injection, hypoglycemia, patient’s socioeconomic status, occupation, and lack of follow-up. Other barriers, such as patient’s age, comorbidities, the difficulty of insulin storage, weight gain, insulin costs, patient’s escape, and insulin unavailability, were relatively scored lower than other barriers.

**Table 1:** General Characteristics of the 45 Doctors and the 203 Patients Included in the Study CKD: chronic kidney disease; CVD: cardiovascular disease; SD: standard deviation; T2DM: type 2 diabetes mellitus

Variable	N (%) or mean ± SD
Doctors	
Age (years)	39.93 ± 7.16
Gender (men)	38 (84.4)
Specialty	
Endocrinologist	11 (24.4)
Internist	24 (53.3)
Family physician	10 (22.2)
Number patients with T2DM/week	
< 10	13 (28.9)
10 - 50	21 (46.7)
> 50	11 (24.4)
Certificate duration (years)	
< 5	27 (60)
5 - 10	8 (17.8)
> 10	10 (22.2)
Patients	
Age (years)	51.53 ± 8.74
Age ≥ 50 years	124 (61.1)
Gender (man)	102 (50.2)
Marital status (married)	183 (90.1)
Residency (urban)	153 (75.4)
Employed	44 (21.7)
Self-employed	74 (36.5)
Unemployed	85 (41.9)
Illiterate	52 (25.6)
Primary	91 (44.8)
Secondary	38 (18.7)
College	22 (10.8)
T2DM duration (years)	5.45 ± 4.14
T2DM duration ≥ 10 years	40 (19.7)
Family history of T2DM	151 (74.4)
Family history of insulin therapy	107 (52.7)
Previous insulin therapy	42 (20.7)
Hypertension	100 (49.3)
Dyslipidemia	132 (65)
CKD	47 (3.9)
CVD	8 (23.2)
Diabetic retinopathy	78 (38.4)
Diabetic neuropathy	132 (65)

Patient-related barriers

The general characteristics of the patients are summarized in Table 1. Among these patients, 42 (20.7%) had a history of insulin therapy, and 31 (73.8%) had decided to discontinue insulin therapy themselves.

**Figure 2:**
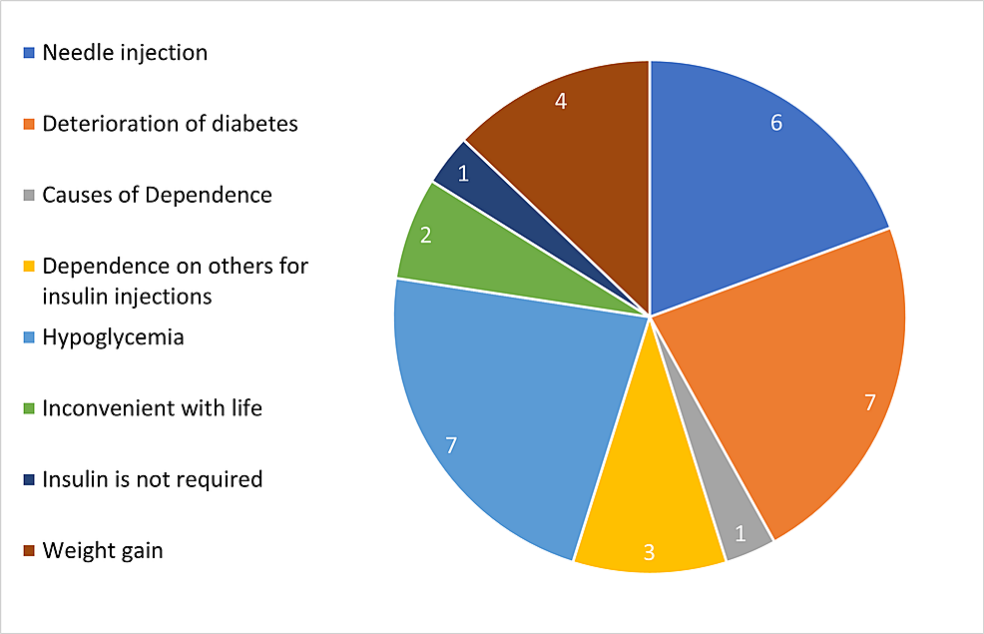
Reasons of insulin discontinuation by the patients with previous insulin experience

The top three reasons for insulin discontinuation were deterioration of diabetes, hypoglycemia, and needle injection, as shown in Figure 2. When the patients were asked about whether they were willing to start insulin therapy if their physician advised it as the only treatment to control diabetes, 99 (48.8%) patients stated that they would initiate therapy, while 104 (51.2%) patients were found to be unwilling.

Illiteracy and primary education were associated with the patients' significantly lower tendency to start insulin therapy, as compared to patients with secondary or college education. Also, patients with a family history of insulin therapy were significantly more likely to accept insulin therapy, as shown in Table 2. Other general and clinical characteristics of the patients were not correlated significantly.

**Table 2:** Correlation of the General and Clinical Characteristics of the 203 Study Patients with Their Willingness to Start Insulin * Percentage within rows ** P-values were calculated using the Chi-square test, except for CKD by Fisher Exact Test CKD: chronic kidney disease; CVD: cardiovascular disease; T2DM: type 2 diabetes mellitus

	Will start insulin*	Will not start insulin*	P-value**
Age ≥ 50 years	61 (49.2%)	63 (50.8%)	0.87
Age < 50 years	38 (48.1%)	41 (51.9%)	
Men	50 (49%)	52 (51%)	0.94
Women	49 (48.5)	52 (51.5)	
Married	92 (50.3%)	91 (49.7%)	0.19
Unmarried	7 (35%)	13 (65%)	
Urban	79 (51.6%)	74 (48.4)	0.15
Rural	20 (40)	30 (60)	
Employed	27 (61.4)	17 (38.6)	0.09
Self-employed	30 (40.5)	44 (59.5)	
Unemployed	42 (49.4)	43 (50.6)	
Illiterate	16 (30.8)	36 (69.2)	0.01
Primary	47 (51.6)	44 (48.4)	
Secondary	23 (60.5)	15 (39.5)	
College	13 (59.1)	9 (40.9)	
T2DM duration ≥ 10 years	77 (47.2)	86 (52.8)	0.37
T2DM duration < 10 years	22 (55.0)	18 (45.0)	
Family history of T2DM	78 (51.7)	73 (48.3)	0.16
No family history of T2DM	21 (40.4)	31 (59.6)	
Family history of insulin therapy	60 (56.1)	47 (43.9)	0.02
No family history of insulin therapy	39 (40.6)	57 (59.4)	
Previous insulin therapy	20 (47.6)	22 (52.4)	0.86
No previous insulin therapy	79 (49.1)	82 (50.9)	
Hypertension	46 (46.0)	54 (54.0)	0.43
No hypertension	53 (51.5)	50 (48.5)	
Dyslipidemia	67 (50.8)	65 (49.2)	0.43
No dyslipidemia	32 (45.1)	39 (54.9)	
CKD**	1 (12.5)	7 (87.5)	0.06
No CKD	98 (50.3)	97 (49.7)	
CVD	18 (38.3)	29 (61.7)	0.10
No CVD	81 (51.9)	75 (48.1)	
Diabetic retinopathy	41 (52.6)	37 (47.4)	0.39
No diabetic retinopathy	58 (46.4)	67 (53.6)	
Diabetic neuropathy	66 (50.0)	66 (50.0)	0.63
No diabetic neuropathy	33 (46.5)	38 (53.5)	

**Figure 3:**
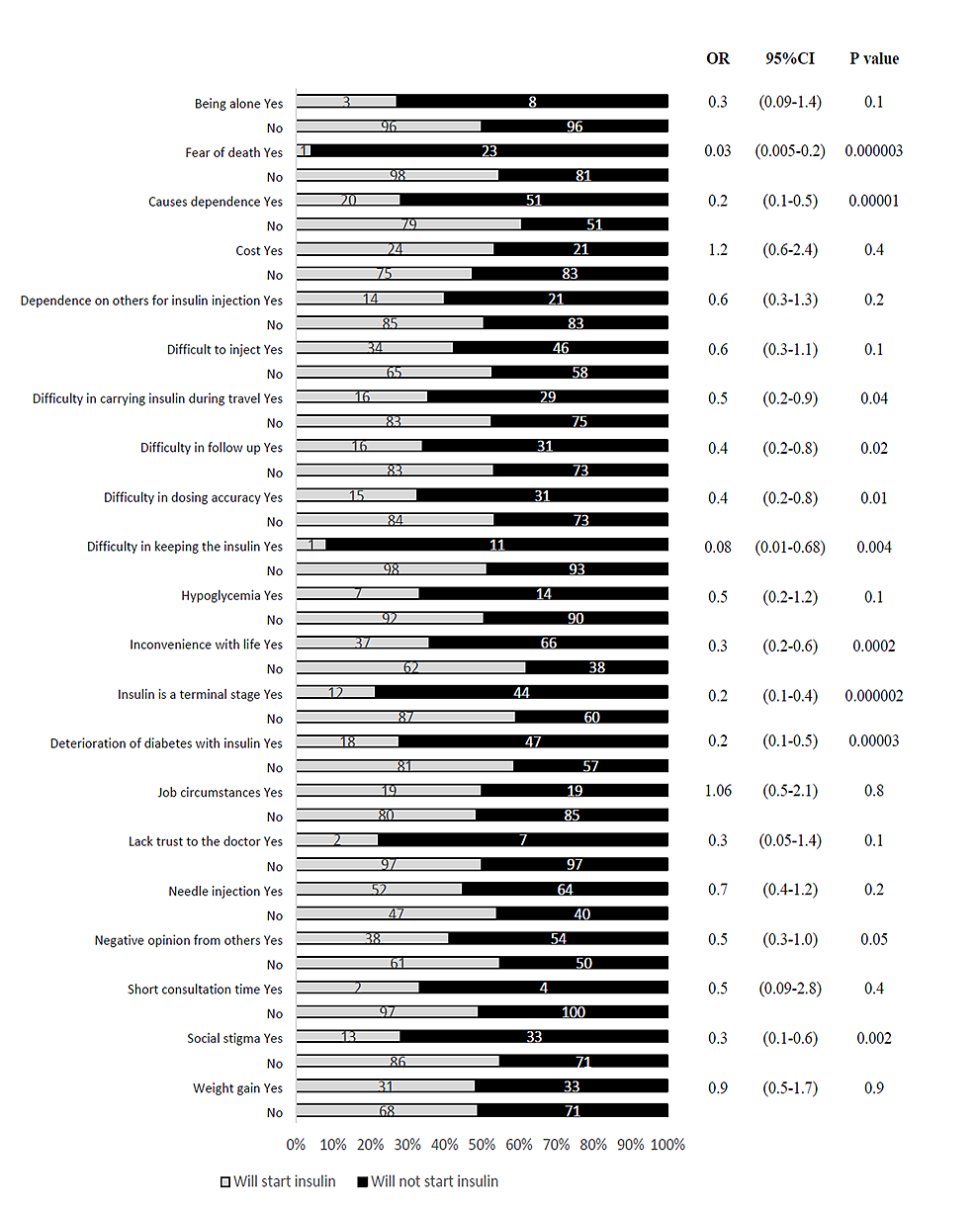
Correlation of the 21 barriers with the patients willing to start insulin CI: confidence interval; OR: odds ratio

Figure 3 shows the correlation between each barrier and the patient’s willingness to start insulin therapy. Among these barriers, fear of death, dependence on others, the difficulty of carrying insulin while traveling, follow-up challenges, the difficulty of dosing accuracy, the difficulty of keeping insulin, inconveniences in daily life, considering insulin therapy as the last resort, deterioration of diabetes with insulin, and viewing insulin therapy as a social stigma were significantly associated with the patients’ lower willingness to start this therapy. The remaining barriers also appeared to have similar but insignificant effects.

## Discussion

The challenges of initiating insulin therapy in T2DM patients are complex due to barriers among both patients and physicians which are usually closely linked. The phenomenon of psychological insulin resistance, in which insulin refusal is based on the patient’s perceptions, fears, or assumptions of therapy (but not clinical facts), can affect physicians, as well as patients [13]. Consequently, when a physician delays insulin initiation, it may strengthen the patient’s belief that the need for insulin represents a major decline in his/her health [12, 14].

In the Delay of Insulin Initiation in Patients with Type 2 Diabetes Mellitus Inadequately Controlled with Oral Hypoglycemic Agents [Analysis of Patient- and Physician-related Factors] (DIPP)-FACTOR study from Korea in 2017, the physician-related barriers included the physicians' concerns about the patients' refusal of treatment, patient compliance, hypoglycemia, lack of disease knowledge, and controllable disease with oral antidiabetic drugs (OADs) [15]. This study also identified less important physician-related barriers, such as the need for follow-up, loss to follow-up, need for dietary changes, inadequate time for discussing therapy, weight gain, the inefficacy of treatment, and comorbidities.

In another study, fear of hypoglycemia, the patient’s refusal of insulin therapy, lack of confidence in physicians, and needle injections were among frequently recognized barriers [16]. Also, insulin affordability, availability, and storage, as well as frequent glucose monitoring, were of great significance.

Another study from Saudi Arabia in 2018 reported that the lack of experience, patient’s refusal of treatment, patient’s non-compliance, patient’s age, need for hospitalization, and fear of hypoglycemia were the most common physician-related barriers [17].

Moreover, Escalada et al. concluded that the physician’s assumption of insulin as the last resort, hypoglycemia, weight gain, considering insulin therapy to be unsafe, and interference in the patient’s social life were significant barriers [18].

Overall, these four studies, along with the present study, showed that the majority of physician-related barriers were linked to patient-related barriers [15-18]. While hypoglycemia and weight gain are well-established side effects of insulin therapy, they were not the most important barriers in these studies.

Regarding patient-related barriers, a family history of insulin use increased the patient's willingness to start insulin, while a low educational level decreased the patient's willingness to start insulin in this study. It seems that the presence of another family member using insulin can help overcome the barriers and increase the patient’s willingness to start insulin therapy. In this regard, Taylor et al. found that negative attitudes toward insulin use were related to the deterioration of T2DM with insulin, family and friends’ concerns, injection pain, hypoglycemic risk, increased dependence on physicians, need for increased efforts, the difficulty of dosing accuracy, weight gain, inconveniences in daily life, health deterioration, and inconveniences in engagement in preferred activities [19].

In a Turkish study, injection-related anxiety, fear of diabetes deterioration, the difficulty of dosing accuracy, dependence on others, social stigma, and reduced quality of life were the most common barriers, resulting in the patient’s resistance to initiate insulin [20]. Multiple studies in the United Kingdom have recognized several misperceptions about insulin use among patients, such as beliefs about insulin ineffectiveness, reduced quality of life, regimen complexity, hypoglycemia, weight gain, and needle injection pain [21-25]. Also, in Saudi Arabia, unwillingness to initiate insulin therapy was investigated, which showed that the patients’ negative attitudes toward insulin therapy initiation were related to lifestyle interference, assuming insulin as the last resort, hypoglycemia, and weight gain concerns [26].

The limitations of this study were the small sample size and not addressing the insulin injections modes, such as syringes or pens.

## Conclusions

This was the first study in our locality evaluating the attitudes toward insulin in both physicians and their T2DM patients. There were various physician- and patient-related barriers to insulin therapy for T2DM. The physician-related barriers mainly involved the patients’ attitudes and perceptions of this therapy. While hypoglycemia and weight gain are well-known side effects of insulin therapy, we found that the most important barriers affecting the patient’s decision to start therapy were related to the impact of treatment on social life and misperceptions about the risks of insulin.
